# A Fluorescence Approach to Assess the Production of Soluble Microbial Products from Aerobic Granular Sludge Under the Stress of 2,4-Dichlorophenol

**DOI:** 10.1038/srep24444

**Published:** 2016-04-14

**Authors:** Dong Wei, Heng Dong, Na Wu, Huu Hao Ngo, Wenshan Guo, Bin Du, Qin Wei

**Affiliations:** 1 School of Resources and Environment, University of Jinan, Jinan 250022, PR China; 2 School of Civil and Environmental Engineering, University of Technology Sydney, Broadway, NSW 2007, Australia; 3 Key Laboratory of Chemical Sensing & Analysis in Universities of Shandong, School of Chemistry and Chemical Engineering, University of Jinan, Jinan 250022, PR China

## Abstract

In this study, a fluorescence approach was used to evaluate the production of soluble microbial products (SMP) in aerobic granular sludge system under the stress of 2,4-dichlorophenol (2,4-DCP). A combined use of three-dimension excitation emission matrix fluorescence spectroscopy (3D-EEM), Parallel factor analysis (PARAFAC), synchronous fluorescence and two-dimensional correlation spectroscopy (2D-COS) were explored to respect the SMP formation in the exposure of different doses of 2,4-DCP. Data implied that the presence of 2,4-DCP had an obvious inhibition on biological nitrogen removal. According to EEM-PARAFAC, two fluorescent components were derived and represented to the presence of fulvic-like substances and humic-like substances in Component 1 and protein-like substances in Component 2. It was found from synchronous fluorescence that protein-like peak presented slightly higher intensity than that of fulvic-like peak. 2D-COS further revealed that fluorescence change took place sequentially in the following order: protein-like fraction > fulvic-like fraction. The obtained results could provide a potential application of fluorescence spectra in the released SMP assessment in the exposure of toxic compound during wastewater treatment.

With the rapid development of chemical compounds production and intensive use of various chemicals in the field of agriculture and industry, the presence of toxic chemical compounds in aqueous solution is becoming a severe environmental and public health problem around the world^1^. As one of typical toxic compounds, chlorinated phenols are produced and discharged by a number of industries, such as mining, metal plating facilities, tanneries, causing a negative impact on biological nitrogen removal in the field of wastewater treatment^2^. Moreover, it is of particular concern that their toxicity could impact sludge physicochemical properties and consequently vary the generation of soluble microbial products (SMPs) in wastewater treatment plant (WWTP)^3^. SMP are soluble organic compounds released during normal biomass metabolism, which are composed mainly of carbohydrates, proteins and humic-like materials and as the major component of residual organic material in secondary effluent^4^.

So far, SMP production of activated sludge system in the presence of toxic compound is of many researchers’ interest. Li *et al.*^5^ investigated the SMP production in the presence of 3′,4′,5-tetrachlorosalicylanilide (TCS) by activated sludge system, implying that the dose of TCS leaded to an increased SMP production. Han *et al.*^6^ evaluated the effect of continuous Zn (II) exposure on organic degradation capability and SMP formation of activated sludge, showing that the SMP content together with its main biochemical constituents increased with influent Zn (II) concentration. However, little information is available regarding to SMP production in aerobic granular sludge system. As a novel biological nitrogen removal process, aerobic granular sludge has a higher biomass concentration, a denser and stronger microbial aggregate structure, and more excellent settling capacity than those of activated sludge^7^. To date, aerobic granular sludge has been successfully applied for treating various high strength organic wastewaters, bioremediation of toxic aromatic pollutants, removal of nutrient and adsorption of heavy metals etc.^8^ The structure of aerobic granular sludge could be characterized into three zones, including the presence of an aerobic outer layer and an anaerobic or anoxic core, facilitates co-existence of nitrifying organisms in the outer layers of the granules, as well as (facultative) anaerobic organisms towards the centre of the granules^9^. Hence, it is likely that the production of SMP in aerobic granular sludge system may be different to that of activated sludge due to the layered structure. Therefore, it is desirable to explore the behavior of SMP formation of aerobic granular sludge under the stress of toxic chemical compounds, which is helpful to promote suitable strategy for the controlling SMP in biological wastewater treatment plant.

Recently, many analysis methods have been used to evaluate the formation of SMP in biological treatment systems, including Fourier transform infrared spectrometry (FTIR), molecular weight (MW), fluorescence spectroscopy, scanning electron microscopy (SEM) and energy dispersive X-ray (EDX) microanalysis^10^. Compared with conventional fluorescence spectroscopy, three-dimensional excitation-emission matrix (3D-EEM) is regarded as a rapid, selective and sensitive monitoring tool to elucidate the compositions of SMP or dissolved organic matter (DOM) samples^11^. Moreover, parallel factor analysis (PARAFAC) coupled with 3D-EEM is developed to quantitative and qualitative analyses of complex mixtures in the field of water or wastewater treatment^12^. Specifically, synchronous fluorescence spectroscopy is successfully applied to determination of multi-component present in the DOM without pre-separation^13^. Additionally, two-dimensional correlation spectroscopy (2D-COS) application in synchronous fluorescence has recently been widely used in some aquatic environments to resolve the overlapped peaks problem by extending spectra along the second dimension^12^. Therefore, it is of a particular interest for providing a basis of fluorescence analysis as a potential monitoring tool for SMP production assessment. However, until now, little information could be found regarding to this point.

Herein, the purpose of this study was to evaluate the production of SMP in aerobic granular sludge system under the stress of 2,4-dichlorophenol (2,4-DCP) by using a fluorescence approach. As one of typical chlorinated phenols compound, 2,4-DCP was selected as a target pollutant in this study, since its wildly application in the production of herbicides in agriculture and greatly threatened human health^14^. A combined use of 3D-EEM, PARAFAC, synchronous fluorescence and two-dimensional correlation spectroscopy (2D-COS) were explored to respect the SMP formation in the exposure of 2,4-DCP. The obtained results could provide a useful approach to describe SMP formation, and identify the corresponding component changes of SMP fractions in the exposure of toxic compound by using fluorescence spectra.

## Experimental

### Parent SBR and synthetic wastewater

Aerobic granular sludge was collected from in a lab-scale sequencing batch reactor (SBR) with a working volume of 17 L. The internal diameter and working height of the SBR were 15 and 150 cm, respectively. The SBR was operated at a cycle of 6 h, comprising of 5 min of influent filling, 25 min of anoxic process, 300 min of aeration, 2 min of settling, and 28 min of effluent and idle. The volumetric exchange ratio and hydraulic retention time (HRT) were controlled at 50% and 12 h, respectively.

The influent high-strength nitrogen wastewater of the reactor were listed as follows: COD (as C_6_H_12_O_6_), 600 mg/L; NH_4_^+^ -N (as NH_4_Cl), 200 mg/L; P (as K_2_HPO_4_) 15 mg/L; CaCl_2_, 40 mg/L; MgSO_4_·2H_2_O, 20 mg/L; FeSO_4_·2H_2_O, 20 mg/L and trace element solution 1.0 ml/L. The influent pH values were adjusted to 7.5 to 7.8 by using NaHCO_3_ and HCl.

### Experimental design

The toxicity batch experiment was conducted in eight beakers to evaluate the effect of 2, 4-DCP on SMP production of aerobic granular sludge, and the working volume of each beaker was 0.5 L. Aerobic granular sludge with main size of 1.5 mm was first collected at the end of aeration process, and washed three times by using deionized water to remove the surface soluble ions. Then, aerobic granular sludge was resuspended into 250 mL deionized water and added into each beaker. Next, 250 mL of synthetic wastewater and the prepared 4-CP stock solution were sequentially added into the batch beakers to achieve the test 2,4-DCP concentrations at 5, 15, 20, 25, 40 and 50 mg/L. As a result, the initial COD and NH_4_^+^ -N concentrations of each beaker were about 300 and 100 mg/L, respectively. The mixed liquor suspended solids (MLSS) concentration in each beaker was controlled at 5.0 g/L. The aeration time and aeration rate were set at 6 h and 0.3 L/min, respectively. As a result, the dissolved oxygen (DO) was controlled above 2 mg/L.

### Fluorescence spectra analysis

3D-EEM spectra of SMP samples were measured by using a luminescence spectrometer (LS-55, Perkin-Elmer Co., USA). EEM of excitation spectra were subsequent scanned from 220–400 at 10 nm increments by varying the excitation wavelength from 280–550 nm at 0.5 nm increments with 10 nm intervals, respectively. Synchronous fluorescence was measured by ranging the excitation wavelengths from 250–550 nm with a constant offset (Δλ) of 60 nm^12^. The scanning speed was set at 1200 nm/min for all the measurements.

### Analytical methods

NH_4_^ + ^-N concentration in the bulk liquid was measured according to the standard method^15^. PARAFAC was performed to interpret the 3D-EEM fluorescence data. PARAFAC analysis was conducted using MATLAB 7.6 (Mathworks, Natick, MA, USA) with the N-way toolbox^16^. 2D-COS was employed to synchronous fluorescence spectra with the increased 2,4-DCP exposure as the external perturbation. More detailed information on the mathematical procedures associated to 2D-COS could be found elsewhere^17^.

## Results and discussion

### Toxicity of 2,4-DCP on biological nitrogen removal

**Figure 1:**
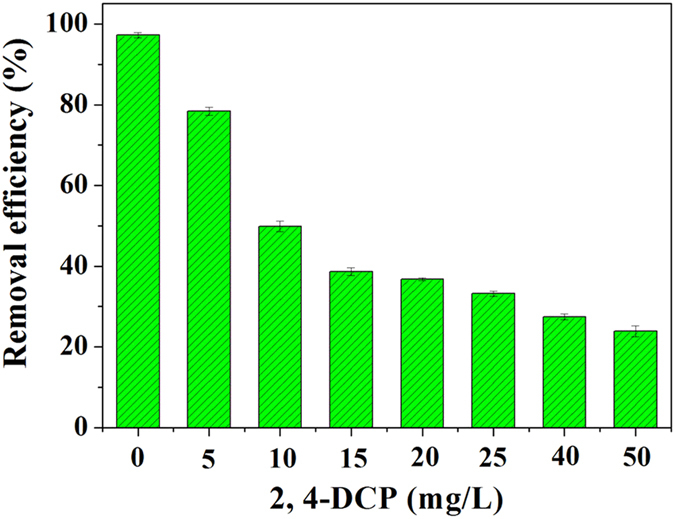
Effect of 2,4-DCP dosage on the variation of nitrogen removal efficiency.

Figure 1 presents the effect of 2,4-DCP dosage on the variation of nitrogen removal efficiency with increased 2,4-DCP concentrations from 0–50 mg/L. Almost 100% NH_4_^+^ -N was removed from aerobic granular sludge system without the presence of 2,4-DCP. However, the effluent NH_4_^+^ -N concentrations increased to 21.6 and 61.3 mg/L in the exposure of 5 and 15 mg/L of 2,4-DCP, corresponding to the removal efficiencies of 78.4 and 38.7%, respectively, which were remarkably lower than that of control experiment. The result suggested that the activity of nitrifying bacteria of aerobic granular sludge was inhibited to the toxicity of 2,4-DCP.

Generally, aerobic and anaerobic granulation processes were two well-known developed biogranulation technologies for wastewater treatment^18^. Compared to conventional activated sludge, biogranulation reactor enables much higher biomass retention and therefore withstands high-strength wastewater and shock loadings. However, regarding to the degradation ability of anaerobic granular sludge, aerobic granular sludge has the poor ability to degradate the wastewater containing toxic compound. Sun *et al.*^19^ found five different phylotypes from responsible for toluene anaerobic degradation, and these included previously identified toluene degraders as well as novel toluene-degrading microorganisms. As a contrast, Shi *et al.*^20^ observed that the presence of tetracycline (TC) had strong toxic effect to nitrifying granules, and led to nitrite accumulation during 60 days operation. However, Carucci *et al.*^21^ successfully developed 4-CP biodegradation in a granulated SBR with acetate as co-substrate, showing good resistance to the toxic effects of high 4-CP concentration even with unacclimated biomass, which was likely related to the high biomass density and to diffusive processes.

### 3D-EEM

**Figure 2:**
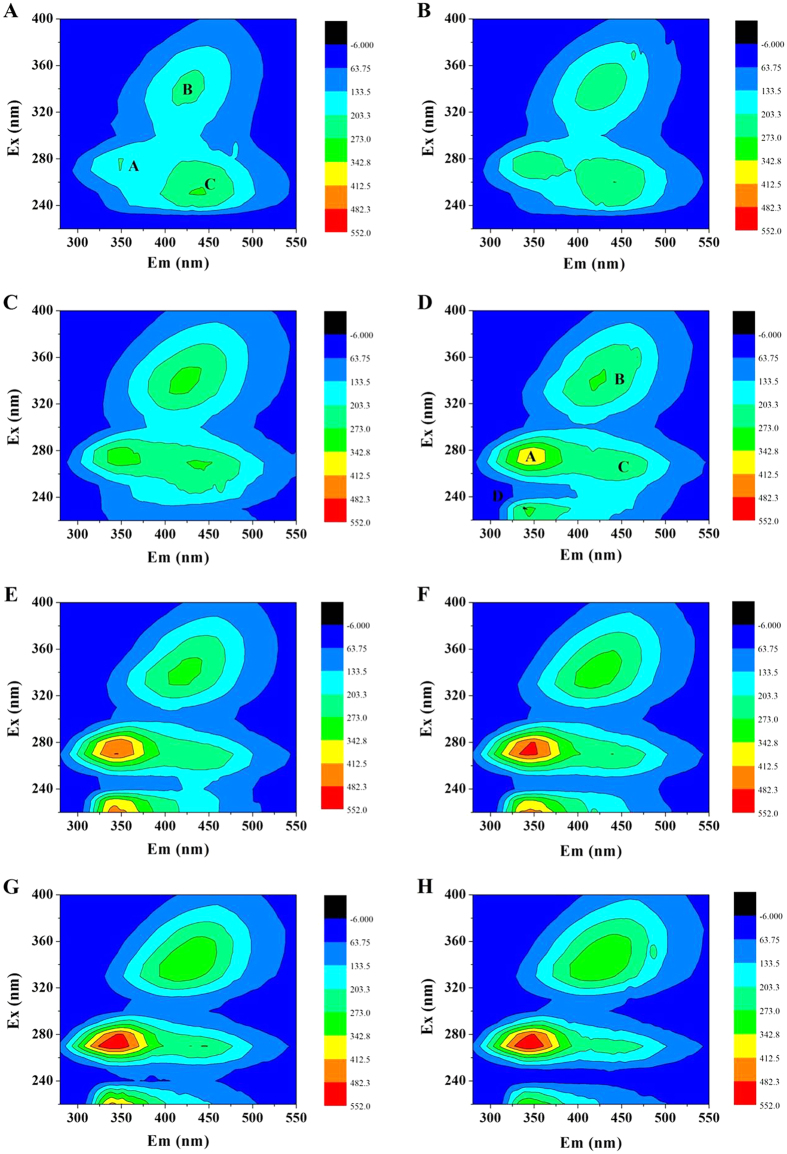
3D-EEM spectra of SMP under various dosages exposure of 2,4-DCP: (**A**) 0 mg/L; (**B**) 5 mg/L; (**C**) 10 mg/L; (**D**) 15 mg/L; (**E**) 20 mg/L; (**F**) 25 mg/L; (**G**) 40 mg/L; (**H**) 50 mg/L.

Figure 2 shows 3D-EEM spectra of SMP samples under various dosages exposure of 2,4-DCP. Table S1 summarizes the fluorescence spectra parameters of SMP samples, including fluorescence location as well as fluorescence peak. Three main fluorescence peaks (Peak A, B and C) were indentified in the SMP sample without the presence of 2,4-DCP exposure. Peak A was located at Ex/Em of 280/350 nm, which was assigned to tryptophan protein-like substances^22^. Peak B and Peak C were indentified at Ex/Em of 340/426.5 and 250/436 nm, which were related to humic acid-like substances and fulvic acid-like substances, respectively^23^.

The presence of 2,4-DCP impacted SMP production from aerobic granular sludge not only fluorescence peak locations but also fluorescence peak intensities. It is evident that fluorescence intensities of Peak A and Peak B increased much higher than that of Peak C in the presence of 2,4-DCP. After 2,4-DCP exposure of 15 mg/L, Peak D was obviously appeared at Ex/Em of 230/345 nm, which was related to aromatic protein-like substances, as similarly reported by Chen *et al.*^23^ The intensities of Peak D also expressed an generally increased trend from 291.1–337.6 with 2,4-DCP concentration. The result suggested that the presence of 2,4-DCP had an important effect on the production of protein -like substances. An obvious blue-shift in terms of excitation wavelength by 10 nm was observed in Peak D, which may be associated with a decomposition of condensed aromatic moieties and the break-up of the large molecules into smaller fragments^24^.

### PARAFAC analysis

**Figure 3:**
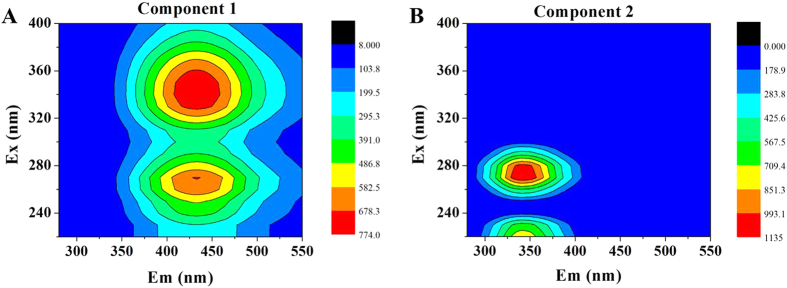
Two components of SMP identified by PARAFAC based on EEM spectra: (**A**) humic-like substances and fulvic-like substances; (**B**) PN-like substances.

In order to deconvolute complex 3D-EEMs into independent fluorescent components which represent groups of similar fluorophores, PARAFAC method was used for quantitative comparison of 3D-EEM samples^25^. Two components of SMP identified by PARAFAC were suitable due to the fact of core consistency diagnostic close to 100%, as displayed in Fig. 3. More detailed, there were two peaks indentified from Component 1, located at Ex/Em of 260/433.5 and 330/435.5, representing to the presence of fulvic-like substances and humic-like substances. Component 2 was similar to peaks previously associated with protein-like substances at Ex/Em of 270/341 and 220/341, respectively.

**Figure 4:**
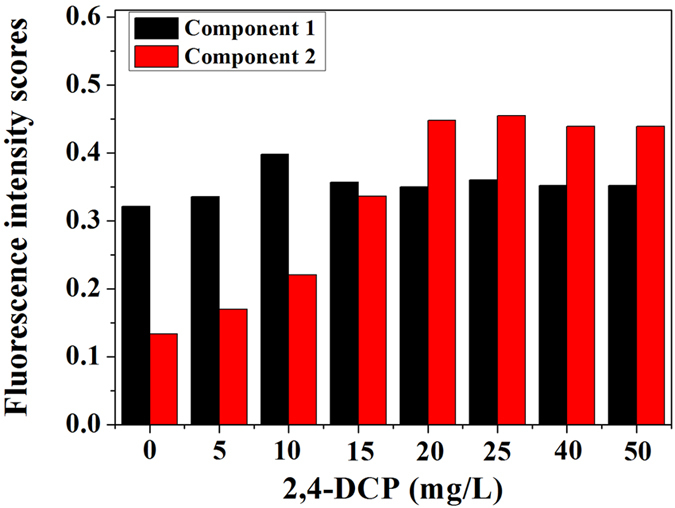
Fluorescence intensity scores of two PARAFAC-derived components in SMP samples as a function of 2,4-DCP concentration.

Figure 4 presents the fluorescence intensity scores of two PARAFAC-derived components in SMP samples as a function of 2,4-DCP concentration. The scores in Component 1 and Component 2 were obvious different to the response of increased 2,4-DCP concentration, which changed from 0.39 and 0.16–0.41 and 0.50, respectively. It demonstrated from EEM-PARAFAC analysis that protein-like substances were more preferentially influenced to the toxicity of 2,4-DCP than other substances.

The application of EEM- PARAFAC for SMP or DOM assessment has been well reported in other literatures. Ni *et al.*^26^ evaluated SMP in the activated sludge process by using EEM-PARAFAC method, implying that two components of fulvic-acid-like substances and humic-like substances were identified based on EEM spectra of the SMP samples. Yu *et al.*^27^ characterized the removal efficiency of DOM in the wastewater treatment plant (WWTP) by using EEM-PARAFAC and second derivative synchronous fluorescence (SDSF), suggesting that SDSF may be a useful tool as PARAFAC for characterizing organic matter in the WWTP performance. The result of this study extended the application of EEM- PARAFAC to the assessment of SMP production in the presence of toxic compound.

### Synchronous fluorescence

**Figure 5:**
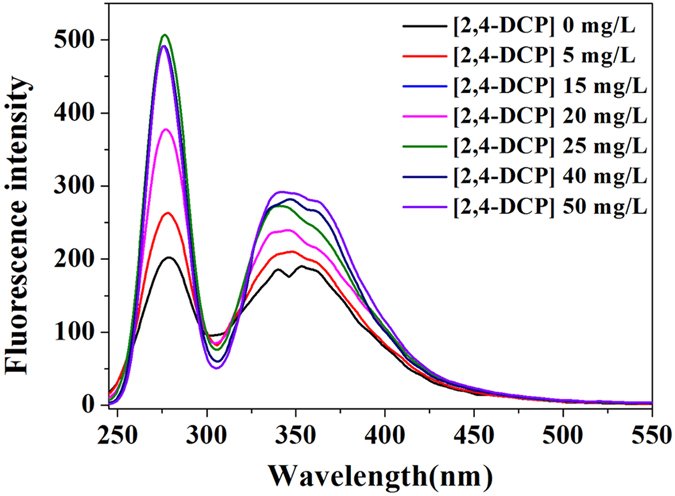
Changes in synchronous fluorescence spectra of SMP under different doses of 2,4-DCP.

Synchronous fluorescence spectroscopy has been successfully applied in multi component analysis, since its high selectivity and sensitivity compared to conventional fluorescence emission or excitation spectral measurement^28^. Figure 5 shows the changes in synchronous fluorescence spectra of SMP under different exposure doses of 2,4-DCP. There were three distinctive regions with the ranges of 250–300, 300–380, and 380–550 nm, assigning to protein-like, fulvic-like, and humic-like fluorescence fractions, respectively^29^. It was obviously shown that fluorescence intensities increased in the whole wavelength upon the increased of 2,4-DCP exposure dosage. Two major peaks, centering at 275 nm and 346 nm, were indentified from synchronous fluorescence spectra. It was found that the protein-like peak presented slightly higher intensity values than that of the fulvic-like peak, which was consistent with the analysis from EEM spectra.

### 2D-COS

**Figure 6:**
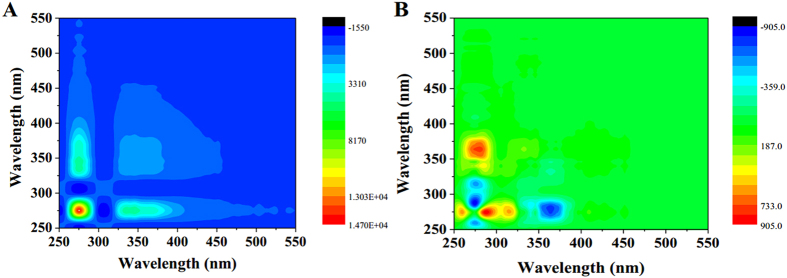
2D-COS maps for the changes of synchronous fluorescence spectra in the presence of 2,4-DCP: (**A**) synchronous map; (**B**) asynchronous map.

2D-COS maps for the changes of synchronous fluorescence spectra in the presence of 2,4-DCP were evaluated and generated a synchronous and an asynchronous map, as displayed in Fig. 6. In this study, the dosage of 2,4-DCP was used as the external perturbation, and thus a set of dosage-dependent fluorescence spectra were obtained. In synchronous map (Fig. 6A), there are two positive autopeaks located at 275 and 346 nm along the diagonal line, indicating that the spectral changes proceed in the same direction for the corresponding areas. The result was consistent with the above increasing synchronous fluorescence intensities upon the addition of 2,4-DCP dosage (Fig. 5). The intensities of autopeaks in this study decreased in the following order: 275 > 346 nm, suggesting that protein-like fraction might be more susceptible to the 2,4-DCP dosage than fulvic-like fraction.

In contrast, asynchronous map reveals the sequential or successive changes of the spectral intensities in response to 2,4-DCP exposure. As shown in Fig. 6B, one obvious positive area and one negative area were observed upper the diagonal line in asynchronous map (Fig. 6B). The positive area was centering at the wavelength pair of 276 nm/363.5 nm, whereas the negative area was centering at the wavelength pair of 276 nm/288 nm. Based on Noda’s rule, fluorescence change took place sequentially in the following order: 288 > 276 > 363.5 nm for SMP. The asynchronous maps demonstrated that protein-like fraction was occurred earlier than that of fulvic-like fraction in the exposure of 2,4-DCP.

## Conclusions

In summary, aerobic granular sludge system was impacted to the exposure of 2,4-DCP during biological nitrogen removal process. The production of SMP was investigated by various analysis methods. 3D-EEM implied the presence of 2,4-DCP impacted SMP production at fluorescence peak locations as well as intensities. Two components of SMP were identified by PARAFAC based on EEM spectra, and the fluorescence intensity score of protein-like substances increased. Two major peaks were indentified from synchronous fluorescence spectra, and protein-like fraction was occurred earlier than that of fulvic-like fraction in the exposure of 2,4-DCP by using 2D-COS.

## Additional Information

**How to cite this article**: Wei, D. *et al.* A Fluorescence Approach to Assess the Production of Soluble Microbial Products from Aerobic Granular Sludge Under the Stress of 2,4-Dichlorophenol. *Sci. Rep.* **6**, 24444; doi: 10.1038/srep24444 (2016).

## Supplementary Material

Supplementary Information
