# Supplementary material for: A Fluorescence Approach to Assess the Production of Soluble Microbial Products from Aerobic Granular Sludge Under the Stress of 2,4-Dichlorophenol
Source: Sci Rep. 2016 Apr 14;6:24444. doi: 10.1038/srep24444 (PMC4830994; doi:10.1038/srep24444)
Supplement: Supplementary Information [file srep24444-s1.pdf]

Supplementary information

A Fluorescence Approach to Assess the Production of Soluble Microbial Products from Aerobic Granular Sludge Under the Stress of 2,4-Dichlorophenol

Dong Wei [a], Heng Dong [a], Na Wu [a], Huu Hao Ngo [b], Wenshan Guo [b], Bin Du [a*], Qin Wei [c]

[a] *School of Resources and Environmental, University of Jinan, Jinan 250022, PR China*

[b] *School of Civil and Environmental Engineering, University of Technology Sydney, Broadway, NSW 2007, Australia*

[c] *Key Laboratory of Chemical Sensing & Analysis in Universities of Shandong, School of Chemistry and Chemical Engineering, University of Jinan, Jinan 250022, PR China*

Dong Wei (E-mail: weidong506@163.com)
Heng Dong (E-mail: dongheng1992@163.com)
Na Wu (E-mail: wuna930201@163.com)
Huu Hao Ngo (E-mail: HuuHao.Ngo@uts.edu.au)
Wenshan Guo (E-mail: Wenshan.Guo-1@uts.edu.au)
Bin Du* (E-mail: dubin61@gmail.com)
Qin Wei (E-mail: sdjndxwq@163.com)

*Corresponding author. Tel. + 86-531-82767370; fax: + 86-531-82765969.
E-mail address: dubin61@gmail.com (Bin Du);

Table S1 Fluorescence spectral parameters of SMP samples under different exposure doses of 2,4-DCP.

2,4-DCP (mg/L)	Peak A		Peak B		Peak C		Peak D	
	Ex/Em	Intensity	Ex/Em	Intensity	Ex/Em	Intensity	Ex/Em	Intensity
0	280/350	204.3	340/426.5	223.9	250/436	278.4	/	/
5	280/350.5	262.7	340/429.5	243.4	260/441.5	273.3	/	/
10	280/350	302.8	340/429.5	293.8	270/435	283.2	/	/
15	280/350	384.8	340/421	282.4	270/444	268.5	230/345	291.1
20	270/343.5	483.2	340/425	298.5	270/441	272.3	220/341.5	446.9
25	270/345	501.9	340/426	341.2	270/439.5	273.9	220/345.5	435.6
40	270/341	542.9	340/423	341.2	270/445.5	276.7	220/340	432.9
50	270/344.5	551.1	340/421	341.5	270/437	252.5	220/345	337.6
